# *Notes from the Field:* Anthrax on a Sheep Farm in Winter — Texas, December 2023–January 2024

**DOI:** 10.15585/mmwr.mm7322a2

**Published:** 2024-06-06

**Authors:** Julie M. Thompson, Kelly Spencer, Melissa Maass, Susan Rollo, Cari A. Beesley, Chung K. Marston, Alex R. Hoffmaster, William A. Bower, Maribel Gallegos Candela, John R. Barr, Anne E. Boyer, Zachary P. Weiner, María E. Negrón, Erin Swaney, Briana O’Sullivan

**Affiliations:** ^1^Division of High-Consequence Pathogens and Pathology, National Centers for Emerging and Zoonotic Infectious Diseases, CDC; ^2^Epidemic Intelligence Service, CDC; ^3^Texas Department of State Health Services; ^4^Division of Laboratory Sciences, National Center for Environmental Health, CDC.

**Summary**

**What is already known about this topic?**

Anthrax is a zoonotic disease. In North America, cases among humans usually follow sporadic animal outbreaks during the hot, dry summer months.

**What is added by this report?**

An unexpected anthrax outbreak occurred during winter in a Texas county adjacent to the Anthrax Triangle, a region with enzootic anthrax. Confirmatory nonculture evidence of *Bacillus anthracis* infection was identified in a lamb and a symptomatic patient who prepared its meat for consumption.

**What are the implications for public health practice?**

Routine anthrax vaccination of animals is needed in this geographic region with known enzootic anthrax. Processing animals that die suddenly from unknown causes should be avoided, irrespective of the season.

Anthrax is a rare but serious infectious zoonotic disease caused by the spore-forming bacterium *Bacillus anthracis*. In North America, animal outbreaks typically occur during summer in hot, dry weather (*1*). Rare cases among humans usually follow direct contact with or processing of anthrax-infected animals or contanimated animal products such as hides, hair, or wool (*1*,*2*). In early 2024, an unusual case of confirmed cutaneous anthrax* acquired during the winter in a geographic region with enzootic anthrax occurred, and an investigation was undertaken. This activity was reviewed by CDC, deemed not research, and was conducted consistent with applicable federal law and CDC policy.^†^

## Investigation and Outcomes

On January 4, 2024, a male rancher aged 50–59 years was evaluated at hospital A for fever, leukocytosis, a black eschar on his right wrist, and extensive edema and blistered lesions on his right arm; he was febrile and had an elevated white blood cell count (Table); anthrax was suspected to be the etiology. Eleven days earlier, on December 24, 2023, he had butchered a lamb that had died suddenly on his ranch, located in a Texas county adjacent to a region with enzootic anthrax, known as the “Anthrax Triangle.”^§^ Before its death, the lamb was healthy and showed no sign of disease. Five persons reported exposure to the lamb. The patient and another person seasoned and cooked the meat; the well-cooked meat was then consumed at a meal with three other persons. Among these five persons, only the index patient exhibited symptoms consistent with cutaneous anthrax, and none experienced symptoms consistent with gastrointestinal anthrax.^¶^

**TABLE:** Timeline of events and diagnostics related to investigation of an anthrax case on a sheep farm in winter — Texas, December 2023–January 2024.

Date	Event	Diagnostic test, sample or source (collection date), location performed	Result	Interpretation
Dec 24, 2023	Lamb death, butchering	**—**	**—**	**—**
Dec 25, 2023	Patient consumed cooked lamb meat	**—**	**—**	**—**
Jan 1, 2024	Patient visited general practitioner	**—**	**—**	**—**
	Cephalexin* 500 mg per os (by mouth) every 8 hrs prescribed			
Jan 4, 2024	Patient developed blisters, edema, and eschar	CBC, blood (Jan 4), Hospital A	Eosinophils count 0 10^3^/*μ*L (Ref = 0–0.40)	Low–normal
	Patient visited Hospital A		Eosinophils percent 0.02% (Ref = 1.00%–5.00%)	Low
	Swabs, serum, and blood cultures collected		Erythrocyte MCH 31.2 pg (Ref = 27.0–31.0)	Normal–high
	Vancomycin^†^ 1 g IV every 24 hrs prescribed		Erythrocyte MCHC 36.0 g/dL (Ref = 33.0–37.0)	Normal–high
	Vancomycin^†^ discontinued		Erythrocyte MCV 86.6 fL (Ref = 80.0–105.0)	Normal
	Ciprofloxacin^§^ 400 mg IV every 8 hrs prescribed		Erythrocyte count 5.21 x 10^6^/*μ*L (Ref = 4.20–6.10)	Normal
	Clindamycin^§^ 600 mg IV every 8 hrs prescribed		Hematocrit 45.1% (Ref = 42.0%–52.0%)	Normal
			Hemoglobin 16.3 g/dL (Ref = 14.0–16.0)	High
			Leukocytes count 14.65 x 10^3^/*μ*L (Ref = 4.80–10.80)	High
			Lymphocytes count 0.43 10^3^/*μ*L (Ref = 1.20–3.40)	Low
			Monocytes count 0.39 10^3^/*μ*L (Ref = 0.10–0.60)	Normal
			Monocytes percent 7.36% (Ref = 1.70%–9.30%)	Normal
			MPV 7.0 fL (Ref = 7.4–10.4)	Low
			Neutrophils count 13.77 10^3^/*μ*L (Ref = 1.40–6.50)	High
			Neutrophils percent 94.01% (Ref = 42.00%–75.20%)	High
			Platelet count 191 x 10^3^/*μ*L (Ref = 130–400)	Normal
Jan 5, 2024	Patient transferred to Hospital B	**—**	**—**	**—**
	Swabs, serum, and blood collected			
Jan 6, 2024	Ewe #1 death, hemorrhage from eyes and nose	**—**	**—**	**—**
Jan 8, 2024	**—**	Culture, patient swab (Jan 5), Hospital B	No growth	**—**
		Real-time PCR,^¶^ patient swab (Jan 6), TX DSHS	Positive	**—**
Jan 11, 2024	Ewe #2 death, hemorrhage from eyes and nose	Culture, patient swab (Jan 6), TX DSHS	No growth	**—**
Jan 12, 2024	Patient discharged	Real-time PCR,^¶^ patient swab (Jan 4), TX DSHS	Positive	**—**
	Swabs collected from both ewes	Culture, patient swab (Jan 4), TX DSHS	No growth	**—**
Jan 15, 2024	Convalescent serum collected from patient	Culture, ewe #1 swab (Jan 12), TVMDL	No growth	**—**
		Culture, ewe #2 swab (Jan 12), TVMDL	No growth	**—**
Jan 30, 2024	**—**	ELISA,** serum (Jan 4), CDC	0 *μ*g/mL	**—**
		ELISA,** serum (Jan 15), CDC	31.4 *μ*g/mL	**—**
Jan 31, 2024	**—**	Mass spectrometry,^††^ serum (Jan 4), CDC	11.9 ng/mL	**—**
		Mass spectrometry,^††^ serum (Jan 15), CDC	Below limit of detection	**—**

**Abbreviations:** CBC = complete blood count; ELISA = enzyme-linked immunosorbent assay; fL = femtoliter (10^−15^ L); HCP = health care provider; IgG = immunoglobulin G; IV = intravenous; MCH = mean corpuscular hemoglobin; MCHC = mean corpuscular hemoglobin concentration; MCV = mean corpuscular volume; MPV = mean platelet volume; pg = picogram (10^−12^ g); PCR = polymerase chain reaction; Ref = reference value or range; TVMDL = Texas A&M Veterinary Medical Diagnostic Laboratory; TX DSHS = Texas Department of State Health Services.

* Cephalosporins are contraindicated for the treatment of naturally occurring *Bacillus anthracis* because of intrinsic resistance.

^†^ Not approved by the Food and Drug Administration for anthrax postexposure prophylaxis or treatment.

^§^ Antimicrobial treatment for systemic anthrax when meningitis has been excluded should include two or more antimicrobial drugs with activity against *B. anthracis:* one or more should have bactericidal activity, and one or more should be a protein synthesis inhibitor.

^¶^ Laboratory Response Network–validated real-time PCR test result considered positive for the presence of *B. anthracis* DNA if all three signatures (BA1, BA2, and BA3) cross the threshold within 40 cycles.

** A more than fourfold rise in anti-protective antigen IgG concentration between the paired acute and convalescent sera is indicative of seroconversion. If the acute serum IgG is ≤3.7 *μ*g/mL, seroconversion is considered to have occured if the convalescent serum result is more than fourfold over 3.7 *μ*g/mL (14.8 *μ*g/mL).

^††^ Total lethal factor activity was analyzed using matrix-assisted laser desorption/ionization time-of-flight mass spectrometry using established, Clinical Laboratory Improvement Amendments–approved analytical methods. These tests have not been cleared or approved by the Food and Drug Administration. The performance characteristics have been established by CDC. The limit of detection is 0.0027 ng/mL. Results for serum are reported in ng/mL.

The patient was initially seen by a general practitioner on January 1 and commenced a course of cephalexin for empiric treatment of soft tissue infection. Anthrax was not initially suspected as the etiology of his symptoms. After 3 days of empiric antibiotic therapy without response, the patient was evaluated at hospital A. A detailed clinical history and the patient’s clinical signs and symptoms raised the index of suspicion for anthrax, and wound swabs and blood were collected before initiation of antimicrobial monotherapy for presumed nonsystemic, cutaneous anthrax. The patient showed signs of systemic involvement and dual therapy for anthrax (ciprofloxacin and clindamycin) was initiated (*3*) the same day. The following morning, he was transferred to hospital B, a larger facility equipped for a more extensive evaluation of his severe edema and malaise. The Texas Department of State Health Services Laboratory performed real-time polymerase chain reaction (PCR) testing and culture from the patient’s wound swabs. Two wound swabs were positive for *B. anthracis* DNA** by real-time PCR; however, culture did not yield an organism consistent with *B. anthracis*. The patient recovered and was discharged after 1 week, on January 12.

The lamb was suspected to be the source of the patient’s illness and, in light of suspected anthrax, interviews were conducted with the patient and his family members. On January 6 and January 11, two ewes subsequently died on the farm with ocular and nasal hemorrhage. Nasal swabs were collected ≥12 hours after death and sent to the Texas A&M Veterinary Medical Diagnostic Laboratory for culture for *B. anthracis*. Test results from both animals were negative; however a high level of clinical and epidemiologic suspicion for anthrax remained. No other animal deaths occurred during the remaining winter season.

Paired sera from the patient were sent to CDC to measure anti-protective antigen (PA) antibodies and lethal factor (LF), a toxin produced by *B. anthracis*, using enzyme-linked immunosorbent assay (ELISA) and mass spectrometry, respectively. A more than fourfold increase in the concentration of anti-PA immunoglobulin G^††^ was noted between serum specimens collected 11 days apart, indicating exposure to *B. anthracis*. LF concentration was 11.9 ng/mL in the acute serum sample,^§§^ one of the highest LF levels ever measured in a patient with cutaneous anthrax at CDC or any other location (*4*). Cooked meat from the lamb was stored frozen for 2 weeks and sent to CDC for real-time PCR and culture. DNA extraction was performed on three separate sections of tissues; all were positive for *B. anthracis* by real-time PCR despite no culture growth.

## Preliminary Conclusions and Actions

Nonculture testing through real-time PCR, ELISA, and mass spectrometry at CDC Laboratory Response Network sites was critical to confirming the diagnosis of anthrax considering of the unusual seasonality and inability to culture *B. anthracis*. Older evidence suggests that first-generation cephalosporins might be effective against *B. anthracis* (*5*) and might have prevented culture growth. However, treatment of naturally occurring *B. anthracis* with cephalosporins is contraindicated because of intrinsic resistance (*3*). This patient recovered only after receiving treatment with antimicrobials effective against anthrax (*3*).

The lack of culture growth from the two ewes could be attributed to factors including delayed sampling, handling, storing, or shipping swabs. *B. anthracis* DNA was detected in cooked meat from the lamb, and there was no culture evidence of viable bacteria from the meat. The infecting bacteria possibly were inactivated when the meat was cooked at high temperatures; however, there is no safe way to prepare meat for human consumption from an animal that has died of anthrax.

This outbreak occurred on a farm adjacent to the Anthrax Triangle in Texas and near the location of a 2019 human cutaneous anthrax case that was associated with an outbreak in animals, which included 25 culture-positive animal cases (*2*). In both the 2019 case and the current case, the patients reported direct skin exposure to animal carcasses, emphasizing the importance of avoiding processing carcasses of animals that unexpectedly die of unknown causes in this region regardless of the season. If animals must be moved, personal protective equipment should be worn. There was no clear history of routine vaccination against anthrax for this herd, or whether the remaining herd was vaccinated after the three animal deaths. Concerns about vaccine-associated adverse events among goats and horses were previously reported in this area (*2*), and routine animal vaccination remains essential in preventing anthrax in animals and subsequent spillover into humans (*1*). 

## Footnotes

* The national standardized case definition accepted in 2018 by the Council of State and Territorial Epidemiologists defines a confirmed case of cutaneous anthrax as including at least one specific or two nonspecific symptoms and signs that are compatible with cutaneous anthrax (a small, painless, pruritic papule on an exposed surface, a vesicle, or a depressed black eschar; edema or erythema; lymphadenopathy; and fever) and confirmatory laboratory criteria (culture and identification from clinical specimens; demonstration of *B. anthracis* antigens in tissues by immunohistochemical staining; evidence of a fourfold rise in antibodies between acute and convalescent sera; detection of *B. anthracis* or anthrax toxin genes by PCR; or detection of lethal factor [LF] in clinical serum specimens by LF mass spectrometry). https://ndc.services.cdc.gov/case-definitions/anthrax-2018/

† 45 C.F.R. part 46.102(l)(2), 21 C.F.R. part 56; 42 U.S.C. Sect. 241(d); 5 U.S.C. Sect. 552a; 44 U.S.C. Sect. 3501 et seq.

§ The “Anthrax Triangle” represents the geographic region of Texas where laboratory-confirmed animal anthrax cases are most frequent and is bounded by the towns of Eagle Pass, Ozona, and Uvalde. Counties represented in this region include Crockett, Edwards, Kinney, Maverick, Sutton, Uvalde, Val Verde, and Zavala counties.

¶ Signs and symptoms of gastrointestinal anthrax include fever and chills, neck swelling, sore throat, hoarseness, painful swallowing, nausea, vomiting, diarrhea, headache, abdominal pain, and abdominal distention. https://www.cdc.gov/anthrax/about/index.html#cdc_disease_basics_symptoms-symptoms

** Laboratory Response Network–validated real-time PCR test result considered positive for the presence of *B. anthracis* DNA if all three signatures (BA1, BA2, and BA3) cross the threshold within 40 cycles.

†† A more than fourfold increase in anti-PA immunoglobulin G (IgG) concentration between the paired acute and convalescent sera is indicative of a seroconversion. If the acute serum IgG is ≤3.7 *μ*g/mL, seroconversion is evident if the convalescent serum result increases more than fourfold over 3.7 *μ*g/mL (14.8 *μ*g/mL).

§§ Total LF activity was analyzed using matrix-assisted laser desorption/ionization time-of-flight mass spectrometry using established, Clinical Laboratory Improvement Amendments–approved analytical methods. These tests have not been cleared or approved by the Food and Drug Administration. The performance characteristics have been established by CDC. The limit of detection is 0.0027 ng/mL. Results for serum are reported in ng/mL.
